# A Novel Non-Invasive Diagnostic Tool for Determining the Subtype of Primary Aldosteronism Using ^68^ Ga Pentixafor PET/CT

**DOI:** 10.1055/s-0045-1814732

**Published:** 2026-01-05

**Authors:** Ashish K. Acharya, Indirani Muthukrishnan Elangovan, Dinesh Kumar Gauthaman, Shelley Simon

**Affiliations:** 1 Department of Nuclear Medicine, Apollo Hospitals, Chennai, Tamil Nadu, India

**Keywords:** Ga-68 Pentixafor PET/CT, primary aldosteronism, CXCR4, adrenal vein sampling, aldosterone-producing adenoma, bilateral adrenal hyperplasia

## Abstract

**Background:**

Primary aldosteronism (PA) may present as either unilateral or bilateral disease. Differentiating unilateral forms—unilateral aldosterone– producing adenoma (APA) and unilateral adrenal hyperplasia—from bilateral forms—bilateral APA, bilateral adrenal hyperplasia (BAH)—is critical, as the management strategies differ. Currently, PA subtyping is performed using adrenal vein sampling (AVS) and computed tomography (CT). However, AVS is invasive and technically demanding, and CT has limited accuracy. CXCR4 (CXC chemokine receptor type 4) expression is higher in APAs than in normal adrenal tissue and non-functional tumors. Pentixafor, a CXCR4-specific ligand labelled with ^68^ Ga, has shown potential for PA subtyping.

**Materials and Methods:**

This prospective observational study included 13 patients with confirmed PA who underwent ^68^ Ga-Pentixafor positron emission tomography/computed tomography (PET/CT) and 13 individuals without PA as controls. Both visual and semi-quantitative analyses were used to classify patients with PA into unilateral or bilateral subtypes.

**Results:**

Among the 13 patients with PA, 10 were diagnosed with unilateral primary aldosteronism (UPA) and three with bilateral primary aldosteronism (BPA) based on ^68^ Ga-pentixafor PET/CT. The mean standardized uptake value (SUV) _max_ , SUV _ratio_ , and adrenal/liver ratios were significantly higher in patients with UPA than in those with BPA and controls. The 10 patients with UPA underwent adrenalectomy, with histopathological and immunohistochemical analysis confirming the diagnosis. All 10 patients achieved complete biochemical remission post-surgery. The three patients with BPA were started on medical therapy and also achieved biochemical remission.

**Conclusion:**

^68^ Ga-Pentixafor PET/CT is a promising non-invasive imaging modality for PA subtyping and may serve as an alternative to AVS in cases where AVS is inconclusive, non-diagnostic, or contraindicated.

## Introduction

Primary aldosteronism (PA) is a major cause of secondary hypertension. It can be classified as unilateral primary aldosteronism (UPA), which includes aldosterone-producing adenoma (APA) and unilateral adrenal hyperplasia (UAH), or bilateral primary aldosteronism (BPA), which includes bilateral APA and bilateral adrenal hyperplasia (BAH). Differentiating between these forms is critical for patient management. Surgical intervention (adrenalectomy) is the treatment of choice for unilateral disease, whereas medical therapy with mineralocorticoid receptor antagonists (e.g., spironolactone) is preferred for bilateral disease. 1

Conventional subtyping methods include computed tomography (CT) and adrenal vein sampling (AVS). 2 However, CT cannot reliably distinguish a non-functioning adenoma from a functioning one, 3 making AVS the gold standard. 2 Despite its accuracy, AVS is invasive, technically difficult, non-standardized, and expensive. 4 This underscores the need for a non-invasive alternative for PA subtyping.

Recent studies have shown that the expression of the chemokine receptor CXC chemokine receptor type 4 (CXCR4) is significantly higher in APA than in normal adrenal tissue and non-functioning tumours. 5 This expression is localized primarily in the zona glomerulosa of the adrenal cortex. Furthermore, studies suggest that Pentixafor—a Ga-68 labelled ligand for CXCR4—may be useful in classifying PA. 6 7

In this study, we evaluated the diagnostic performance of ^68^ Ga-Pentixafor PET/CT in subtyping PA, with histopathology and immunohistochemistry serving as the reference standards.

## Materials and Methods

This prospective study was conducted over 24 months (January 2023–January 2025) at our institute and included 13 patients with PA and 13 control patients who underwent ^68^ Ga-pentixafor PET/CT for unrelated indications. The primary objective was to evaluate the diagnostic utility of ^68^ Ga-Pentixafor PET/CT in PA subtyping and its potential to guide clinical management.

Inclusion criteria were clinical suspicion of PA (e.g., resistant hypertension); biochemical evidence, including plasma aldosterone >16 ng/dL, serum potassium <3.5 mEq/L, aldosterone-to-renin ratio (ARR) >30 ng/dL/ng/mL/h, and unsuitability for AVS due to comorbidities, age, or patient refusal.

## Methodology

Patients diagnosed with PA underwent ^68^ Ga-pentixafor PET/CT. The radiotracer was synthesized in-house using an automated system (ITG module) and GMP-compliant disposable kits (ABX). Pentixafor (25 µg) was labelled with ^68^ Ga and purified to >98% radiochemical purity, verified using thin-layer chromatography. Patients received 1.85 MBq/kg (0.05 mCi/kg) intravenously. Imaging was performed 30–40 minutes post-injection on a Siemens Biograph Vision 450 digital PET/CT scanner.

**Fig. 1:**
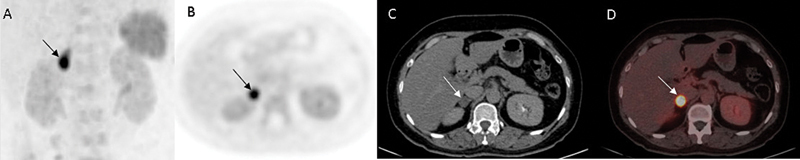
A 47-year-old female with resistant hypertension, recurrent hypokalemia ( *K ^+^* : 2.0 mEq/L), muscle weakness, increased aldosterone of 35 ng/dL, and ARR of 382. ( **A** ) MIP image, ( **B** ) PET, ( **C** ) CT; ( **D** ) Fused image of ^68^ GaPentixafor PET/CT showed focal increased tracer uptake (SUV _max_ – 21.6) in a well-defined heterodense nodule in the lateral limb of the right adrenal gland—suggestive of unilateral APA. APA, aldosterone-producing adenoma; ARR, aldosterone-to-renin ratio; CT, computed tomography; PET, positron emission tomography; MIP, maximum intensity projection; SUV, standardized uptake value.

1. A unilateral adrenal nodule detected on CT with focal increased tracer uptake was considered indicative of unilateral APA ( Figs. 1 2 3 ). Diffuse unilateral increased tracer uptake without a demonstrable CT nodule was considered UAH. Both unilateral APA and UAH were classified under UPA, as the management of both conditions is surgical.
2. Diffuse bilaterally increased tracer uptake (with or without nodules) was considered BPA. This included both BAH, characterized by the absence of nodules ( Fig. 4 ), and bilateral APA, where bilateral CT-detected nodules showed increased tracer uptake. Since the management of both is medical, further subcategorization was not needed.
3. For UPA, the maximum standardized uptake value (SUV _max_ ) was measured by drawing a region of interest (ROI) on the dominant adrenal gland. For BPA, ROIs were drawn on both adrenal glands to obtain two SUV _max_ values.
4. Patients diagnosed with UPA on Ga-68 Pentixafor PET/CT underwent adrenalectomy, whereas those diagnosed with BPA were started on medical management with aldosterone antagonists such as spironolactone and/or eplerenone.
5. Histopathological and immunohistochemical analyses of the patients who underwent surgery were performed to confirm the presence of APA or UAH ( Fig. 5 ).

**Fig. 2:**
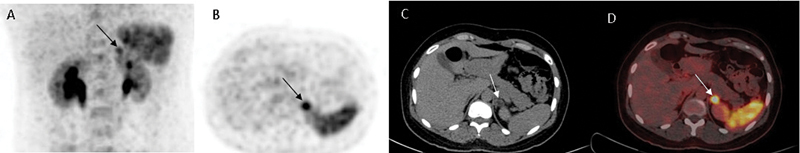
A 35-year-old female with resistant hypertension, hypokalemia ( *K ^+^* = 2.7 mEq/L), increased aldosterone: 21 ng/dL, and ARR: 52. ( **A** ) MIP image; ( **B** ) PET; ( **C** ) CT; ( **D** ) Fused image of ^68^ GaPentixafor PET/CT showed focal increased ^68^ GaPentixafor uptake in a well-defined heterodense nodule in the lateral limb of the left adrenal gland – suggestive of unilateral APA. APA, aldosterone-producing adenoma; ARR, aldosterone-to-renin ratio; MIP, maximum intensity projection; PET/CT, positron emission tomography/computed tomography.

**Fig. 3:**
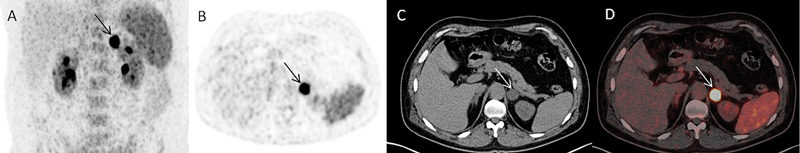
A 45-year-old male with uncontrolled hypertension, hypokalemia ( *K ^+^* : 2.6 mEq/L), increased aldosterone: 48.1 ng/dL, ARR: 436. ( **A** ) MIP image; ( **B** ) PET; ( **C** ) CT; ( **D** ) Fused image of ^68^ GaPentixafor PET/CT showed focal increased ^68^ Ga Pentixafor uptake in a well-defined hypodense nodule in the lateral limb of the left adrenal gland – suggestive of unilateral APA. APA, aldosterone-producing adenoma; ARR, aldosterone-to-renin ratio; MIP, maximum intensity projection; PET/CT, positron emission tomography/computed tomography.

**Fig. 4:**
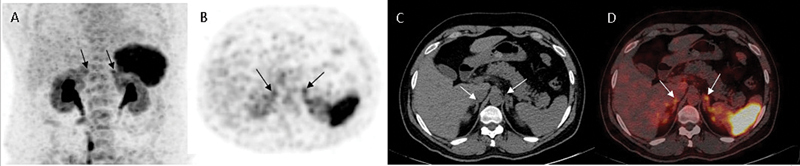
A 45-year-old male with resistant hypertension, hypokalemia ( *K ^+^* : 2.9 mEq/L), increased aldosterone: 28 ng/dL, ARR: 253. ( **A** ) MIP image; ( **B** ) PET; ( **C** ) CT; ( **D** ) fused image of ^68^ Ga-Pentixafor PET/CT showed symmetrically increased diffuse ^68^ Ga CXCR4 uptake in the bilateral adrenals, with focal hypodense nodular thickening in the posterior limb of the left adrenal suggestive of BPA. APA, aldosterone-producing adenoma; ARR, aldosterone-to-renin ratio; BPA, bilateral primary aldosteronism; MIP, maximum intensity projection; PET/CT, positron emission tomography/computed tomography.

**Fig. 5:**
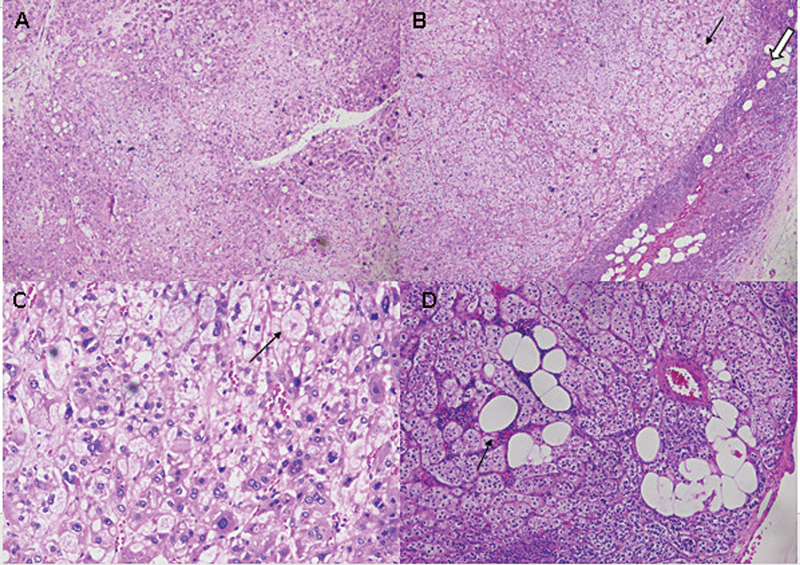
( **A** ) Adrenal adenoma with larger cells having distinct cell borders and abundant foamy cytoplasm; ( **B** ) Adenoma on the left ( *arrow* ) and normal adrenal on the right ( *thick arrow* ); ( **C** ) Eosinophilic inclusion body; ( **D** ) Lipomatous metaplasia ( *arrow* ).

For UPA cases, follow-up was done 6 months after surgery. Patients with BPA were followed up 9 to 12 months after the initiation of medical management. Biochemical remission was assessed using the Primary Aldosteronism Surgical Outcome (PASO) criteria, including normalization of blood pressure, serum potassium, aldosterone, and plasma renin levels. 8

### Statistical Analysis

All analyses were performed using MedCalc v22.023. A *p* -value <0.05 was considered statistically significant. ROC analysis was conducted for SUV _max_ values across control, UPA, and BPA groups.

## Results

The study included 13 participants of Asian origin, comprising six females and seven males, with a mean age of 51 years (range 35–67 years). Among the 13 patients with biochemically confirmed PA, 10 were categorized as having UPA with APA, and three were categorized as having BPA with features of BAH ( Table 1 ).

**Table 1:** Demographic, biochemical, and imaging features of the patients in the study

Patient	Age	Diagnosis	SUV _max_ of the adrenal gland	Size of UPA in maximum dimension (in cm)	S. Aldosterone (ng/dL)	ARR
1	47	UPA	21.6 (Right)	1.3	35	382
2	58	UPA	7.8 (Left)	0.9	28	253
3	67	UPA	8 (Left)	1.0	39	406
4	48	UPA	8.4 (Right)	0.9	32	264
5	45	UPA	35.4 (Left)	1.4	48	436
6	45	BPA	4.9 (Left) 4.3 (Right)	–	28	253
7	56	UPA	12.6 (Left)	0.8	24	46
8	55	BPA	6.7 (Right) 5.5 (Left)	–	40	408
9	65	UPA	5.1	0.9	26	48
10	52	UPA	19.3	1.0	62	284
11	62	UPA	11.8	1.1	570	491
12	35	UPA	7.5 (Left)	0.7	21	52
13	52	BPA	6.2 (Right) 6.4 (Left)	–	24	98

Abbreviation: ARR, aldosterone-to-renin ratio; BPA, bilateral primary aldosteronism; SUV, standardized uptake value; UPA unilateral primary aldosteronism.

The mean SUV _max_ in patients with UPA was 13.7 (range: 5.1–35.4), which was significantly higher than in patients with BPA (5.7 [range: 4.3–6.7]) and control patients (2.9 [range: 1.8–4.1]). The SUV _ratio_ , calculated as the ratio of the SUV _max_ of the dominant adrenal to that of the contralateral adrenal, had a mean value of 5.1 in patients with UPA, compared with 1.1 in both BPA and control patients. The adrenal-to-liver ratio, defined as the ratio of the SUV _max_ of the dominant adrenal to the SUV _max_ of the liver, was 4.2 in patients with UPA, 1.9 in patients with BPA, and 0.9 in controls ( Table 2 ).

**Table 2:** Mean SUV _max_ , mean SUV _ratio_ , and mean adrenal/liver ratio of UPA, BPA, and control cases

Mean	SUV _max_	SUV _ratio_	Adrenal/Liver ratio
UPA	13.7	5.1	4.2
BPA	5.7	1.1	1.9
Control	2.9	1.1	0.9

Abbreviations: BPA, bilateral primary aldosteronism; SUV, standardized uptake value; UPA, unilateral primary aldosteronism.

All 10 patients diagnosed with UPA underwent adrenalectomy. Histopathological and immunohistochemical analyses confirmed the presence of APA in each case. All patients achieved complete biochemical remission following surgery.

The three patients with BPA were initiated on medical management, and biochemical remission was achieved during follow-up.

**Fig. 6:**
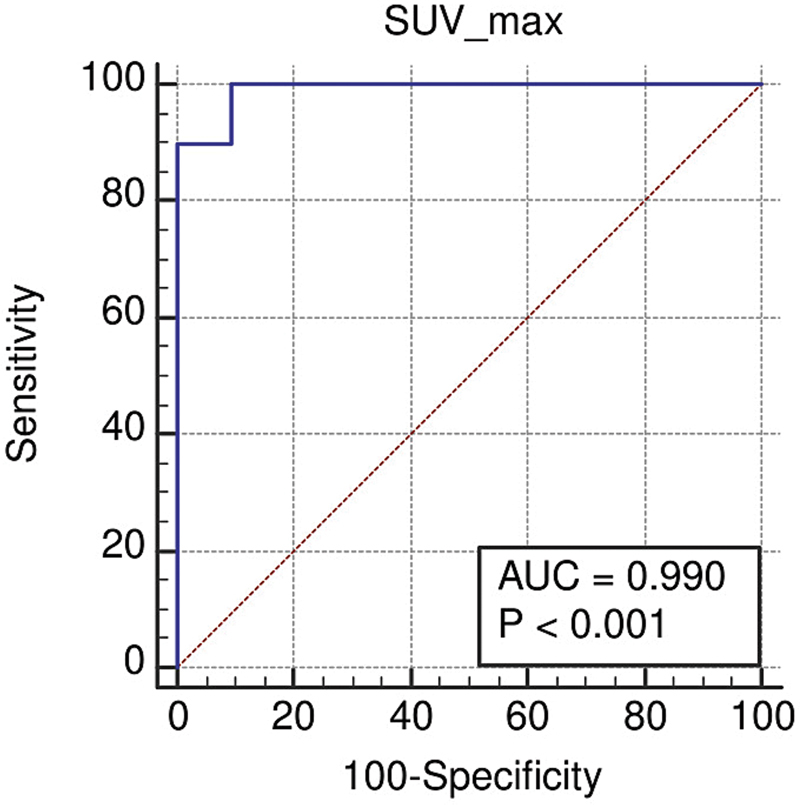
Receiver operating characteristic (ROC) curve.

The optimal cut-off value for differentiating UPA from BPA and control cases was >4.9, with a 95% confidence interval (CI) of 3.8 to 6.7. The area under the curve (AUC) was 0.990 (95% CI: 0.914–1.000), indicating excellent discriminatory capability ( Fig. 6 ). The Youden index was 0.904, reflecting strong overall diagnostic performance. Sensitivity was 100% (95% CI: 69.2–100%), and specificity was 90.48% (95% CI: 77.4–97.3%). The positive likelihood ratio (LR + ) was 10.50, indicating a high probability of disease when the test result was positive. Conversely, the negative likelihood ratio (LR–) was 0.11, suggesting that a negative test result significantly reduced the likelihood of disease ( Table 3 ).

**Table 3:** SUV _max_ cut-off for differentiating UPA from BPA and control cases

SUV max Cut-off (95% CI)	Area under the curve (AUC)	Youden index	Sensitivity	Specificity	Positive LR	Negative LR
>4.9 (3.8–6.7)	0.990 (0.914–1.000)	0.904	100% (69.2–100%)	90.48% (77.4–97.3%)	10.50	0.11

Abbreviations: BPA, bilateral primary aldosteronism; LR, likelihood ratio; SUV, standardized uptake value; UPA, unilateral primary aldosteronism.

The study evaluated the diagnostic accuracy of the SUV _ratio_ , establishing a cut-off value of >1.4 for UPA, with a 95% CI of 1.2 to 1.4. The AUC was 1.00 (95% CI: 0.868–1.000), indicating perfect discriminative ability. The Youden index was 1.000, reflecting optimal test performance. Sensitivity and specificity were both 100% (95% CI for sensitivity: 69.2–100% and for specificity: 79.4–100%), demonstrating exceptional diagnostic accuracy. The LR+ was 16.00, signifying a strong association with disease presence when the test was positive. The LR– was 0.10, indicating that a negative result significantly reduced the probability of disease.

The study also assessed the diagnostic accuracy of the adrenal-to-liver SUV _ratio_ , identifying a cut-off value of >2.0 for UPA, with a 95% CI of 1.8 to 2.0. The AUC was 0.988 (95% CI: 0.845–1.000), demonstrating excellent discriminative capability. The Youden index was 0.900, indicating strong overall test performance. Sensitivity was 90% (95% CI: 55.5–99.7%) and specificity was 100% (95% CI: 79.4–100%), ensuring high diagnostic reliability. The LR+ was 7.20, suggesting a strong probability of disease when the test was positive. Conversely, the LR– was 0.11, indicating that a negative test result significantly reduced the likelihood of disease.

**Fig. 7:**
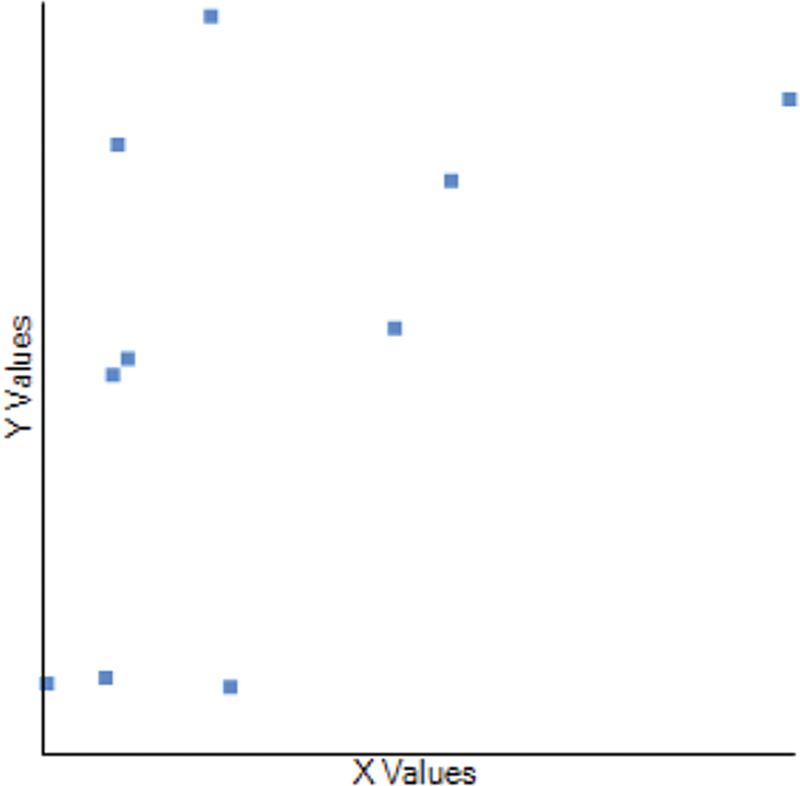
Scatter plot for Pearson correlation co-efficient between SUV _max_ of UPA and ARR. The X- axis shows the SUV _max_ values of UPA and the Y-axis shows the ARR of the patients. ARR, aldosterone-to-renin ratio; SUV, standardized uptake value; UPA, unilateral primary aldosteronism.

The study also evaluated the relationship between the SUV _max_ of UPA and the biochemical parameter, i.e., ARR. Pearson correlation co-efficient calculator only showed a weak relationship between the two variables ( *R* = 0.49, *p* -value of 0.14) ( Fig. 7 ).

## Discussion

Hu et al 9 conducted a study on 100 patients with PA, of whom 43 were diagnosed with APA and 57 with BAH. They observed that the aldosterone–cortisol ratio in adrenal veins positively correlated with adrenal SUV _max_ at 10 minutes post-PET/CT. The lateralization index derived from SUV _max_ at 10 minutes yielded an AUC of 0.90. A cut-off value of 1.65 for the lateralization index had a specificity of 1.00 and a sensitivity of 0.77. The concordance rate between PET/CT and AVS was 90%, compared to only 54% between conventional CT and AVS.

Heinze et al 5 demonstrated that tracer uptake was significantly higher on the side of increased aldosterone secretion in patients with APA when imaged with Ga-68 Pentixafor PET/CT. An SUV _max_ cut-off value of 4.9 provided a sensitivity of 88.9% and specificity of 87.2%. Higher cut-offs such as 7.3 achieved 100% specificity (with 77.8% sensitivity), and 4.7 achieved 100% sensitivity (with 83.7% specificity). The AUC for this study was 0.964.

Ding et al 10 studied 123 patients with adrenal micronodules using ^68^ Ga-Pentixafor PET/CT. The sensitivity, specificity, and overall accuracy for identifying patients eligible for surgery were 90.2, 72.7, and 86.5%, respectively—significantly higher than those of adrenal CT. The concordance rate between AVS and PET/CT was 66.7%. Furthermore, PET/CT correlated more strongly with surgical outcomes compared with AVS (82.4 vs. 68.86%). Their semi-quantitative diagnostic thresholds for surgically treatable lesions were an SUV _max_ of 4.55, a lesion-to-liver SUV _ratio_ of 2.17, and a lesion-to-normal adrenal SUV _ratio_ of 1.90. All patients showed clinical benefit following the removal of Ga-68Pentixafor-avid lesions.

Our study findings are consistent with those of the aforementioned studies and reinforce the utility of Ga-68 Pentixafor PET/CT in the evaluation and management of PA. The high sensitivity and specificity observed in our cohort may be attributed to the use of histopathological and immunohistochemical examination (HPE & IHC) as the diagnostic gold standard, rather than AVS. Our study also demonstrated a higher mean SUV _max_ in UPA (13.7) compared to BPA (5.7). The semi-quantitative thresholds for surgical candidacy in our study were an SUV _max_ of 4.9, an adrenal-to-liver SUV _ratio_ of 2.0, and a lesion-to-normal adrenal SUV _ratio_ of 1.4.

Notably, prior studies varied in the timing of PET/CT acquisition post-Ga-68 Pentixafor injection, ranging from 10 to 60 minutes, with most scans acquired between 30 and 45 minutes. 5 9 10 11 12 13 14 Based on this, we standardized our imaging protocol to 30–40 minutes post-injection. Future studies involving dual- or multi-timepoint imaging may help determine the optimal acquisition window, accounting for tracer washout kinetics.

Current guidelines suggest that patients with suppressed renin levels (plasma renin activity <1.0 ng/mL/h) should be presumed to have PA until proven otherwise. According to Endocrine Society recommendations, all hypertensive patients should be screened for PA at least once, given the increased risk of end-organ damage associated with PA compared with other hypertension etiologies. Since Ga-68 Pentixafor PET/CT is still in the early phase of clinical application, our inclusion criteria focused on patients with a higher aldosterone-renin ratio (>30), which may have contributed to the high diagnostic accuracy in this study.

None of the 10 patients with UPA were found to have UAH on final histopathology. This may be attributed to the significantly lower incidence of UAH (approximately 2%) among PA cases. The three patients with BPA had either normal appearing adrenal glands or minimal nodular thickening and were categorized as BAH. However, early-stage bilateral APA could not be completely ruled out. As both BAH and bilateral APA require the same management approach (i.e., medical management), further categorization was unnecessary.

A notable limitation of this study was its small sample size of only 13 patients, which restricts statistical power and limits generalizability. Larger-scale investigations encompassing a more extensive patient cohort are imperative to yield more robust and precise results, facilitating a deeper understanding of the diagnostic utility of Ga-68 Pentixafor PET/CT in subtyping PA. Thus, future studies with expanded sample sizes are warranted to validate and augment the observations made in this study, thereby enhancing the clinical applicability and reliability of the findings.

## Conclusion

Ga-68 Pentixafor PET/CT is a promising, non-invasive imaging modality for subtyping PA. It may serve as an alternative or adjunct to AVS, particularly when AVS is inconclusive, non-diagnostic, or contraindicated. The technique has demonstrated high sensitivity and specificity in detecting and differentiating PA subtypes.
